# Transmission of Hepatitis E Virus in Developing Countries

**DOI:** 10.3390/v8090253

**Published:** 2016-09-20

**Authors:** Mohammad S. Khuroo, Mehnaaz S. Khuroo, Naira S. Khuroo

**Affiliations:** 1 Sher-I-Kashmir Institute of Medical Sciences, Srinagar, Kashmir 190001, India; 2 Digestive Diseases Centre, Dr. Khuroo’s Medical Clinic, Srinagar, Kashmir 190010, India; naira_sultan@yahoo.com; 3 Department of Pathology, Government Medical College, Srinagar, Kashmir 190001, India; mkhuroo@yahoo.com

**Keywords:** hepatitis E virus, hepatitis E, transmission, zoonosis

## Abstract

Hepatitis E virus (HEV), an RNA virus of the *Hepeviridae* family, has marked heterogeneity. While all five HEV genotypes can cause human infections, genotypes HEV-1 and -2 infect humans alone, genotypes HEV-3 and -4 primarily infect pigs, boars and deer, and genotype HEV-7 primarily infects dromedaries. The global distribution of HEV has distinct epidemiological patterns based on ecology and socioeconomic factors. In resource-poor countries, disease presents as large-scale waterborne epidemics, and few epidemics have spread through person-to-person contact; however, endemic diseases within these countries can potentially spread through person-to-person contact or fecally contaminated water and foods. Vertical transmission of HEV from infected mother to fetus causes high fetal and perinatal mortality. Other means of transmission, such as zoonotic transmission, can fluctuate depending upon the region and strain of the virus. For instance, zoonotic transmission can sometimes play an insignificant role in human infections, such as in India, where human and pig HEV infections are unrelated. However, recently China and Southeast Asia have experienced a zoonotic spread of HEV-4 from pigs to humans and this has become the dominant mode of transmission of hepatitis E in eastern China. Zoonotic HEV infections in humans occur by eating undercooked pig flesh, raw liver, and sausages; through vocational contact; or via pig slurry, which leads to environmental contamination of agricultural products and seafood. Lastly, blood transfusion-associated HEV infections occur in many countries and screening of donors for HEV RNA is currently under serious consideration. To summarize, HEV genotypes 1 and 2 cause epidemic and endemic diseases in resource poor countries, primarily spreading through contaminated drinking water. HEV genotypes 3 and 4 on the other hand, cause autochthonous infections in developed, and many developing countries, by means of a unique zoonotic food-borne transmission.

## 1. Introduction

Hepatitis E is an enterically transmitted, self-limiting, acute, viral hepatitis [1]. The disease is ecologically dependent and is a major public health concern, especially in resource-poor countries [2]. The disease causes large-scale, water-borne epidemics of viral hepatitis and is the most common cause of acute sporadic hepatitis and fulminant hepatic failure in such countries [3]. The disease has unique and as yet unexplained epidemiological characteristics, including repeated waves of large-scale epidemics, occurrence of disease in adult population and high incidence and severity of disease in pregnant women [4]. The infection is prevalent in a wide range of animal species and human zoonotic hepatitis E is encountered in many developing countries and in all industrialized countries [5,6]. This paper shall focus its review on the mode of transmission of hepatitis E virus in developing countries [7].

Hepatitis E was discovered when a massive waterborne outbreak of jaundice was discovered, which had hit Gulmarg region, Kashmir, India in November 1978, and was classified as an “epidemic non-A, non-B hepatitis” [8]. The region had very hard weather conditions, inadequate primary healthcare, and poor access to tertiary care. Investigating this epidemic has been a remarkable human interest story [9], which unraveled unprecedented clinical and epidemiological data and led to postulating the existence of another human hepatitis virus [4,10,11,12,13,14]. Simultaneously, a disease with similar clinical features causing around one-half of endemic hepatitis was identified [15,16,17,18,19]. The virus-like particles (VLP) were identified using immune electron microscopy (IEM) from stool samples collected on days 28, 43, 44, and 45 from a volunteer who self-ingested extracts of nine fecal samples from patients with an epidemic of non-A, non-B hepatitis, affecting Soviet military personal in Afghanistan [20]. Over the next seven years, animal transmission studies and extensive physicochemical properties of the putative agent were performed [21]. Finally, cloning and partial sequencing of the virus recovered from the bile of the macaque was reported [22]. Soon afterwards, full length sequencing of the virus (7.2 kb) [23] and serological tests for diagnosis were readily available [24].

## 2. Overview of Hepatitis E Virus (HEV)

Hepatitis E virus (HEV) is one of the five major hepatotropic viruses, which primarily affect the liver [25]. HEV infection in humans causes acute hepatitic illness, designated as hepatitis E [4]. HEV is an RNA virus, spherically shaped, non-enveloped, and icosahedral in symmetry, with surface spikes and indentations [26]. The diameter of the virion is 27 to 32 nm on IEM, 32 to 34 nm after sucrose gradient centrifugation, and 38.5 to 42 nm on cryo-electron microscopy (cryo-EM) analysis. HEV has marked heterogeneity and hepatitis E-like viruses have been isolated and sequenced from a number of animals, including domestic pig, wild boar, Sicca deer, moose, rabbit, dromedary, chicken, bats, ferret, mink, rats, mongoose, and cutthroat trout [5,6,27]. All hepatitis-like viruses have been classified as members of *Hepeviridae* family [28] and divided into two genera, namely *Orthohepevirus* and *Piscihepevirus*. *Orthohepevirus* has four species: A (all mammalian isolates, such as human, pig, wild boar, deer, mongoose, rabbit, and camel); B (all 3 avian isolates, such as avian HEV-1, avian HEV-2, and avian HEV-3); C (isolates from rats, greater bandicoot, Asian musk shrew, and ferrets) and D (isolates from bats). *Piscihepevirus* includes a single isolate from cutthroat trout. Mammalian HEVs include several genotypes that can infect specific animals differently—for instance, genotypes HEV-1 and HEV-2 only infect humans, while genotypes HEV-3 and HEV-4 infect domestic pigs, wild boar, deer, mongoose, and humans (rabbit HEV is a distant member of genotype HEV-3 [29,30]). Lastly, genotype HEV-7, designated as DcHEV, primarily infects dromedaries. However, it has recently been found to have zoonotic potential when an infected liver transplant patient from the Middle East was discovered to have obtained HEV after consuming camel flesh and drinking camel milk [31,32]. Several animals, including the rhesus macaque, the cynomolgus monkey, horses, goat, sheep, cattle, and dogs show serological evidence of HEV infection; however, HEV strains from such animals need further characterization [33].

## 3. Hepatitis E in Developing Countries

Hepatitis E in developing countries has several disease patterns, determined by the geographical distribution of HEV genotypes, socioeconomic conditions, level of sanitation, access to potable water, and regional occurrence of zoonotic HEV infections in the animals [34,35,36].

### 3.1. HEV-1 and HEV-2

HEV-1 and HEV-2 exclusively infect humans and lack any zoonotic origin [27]. Global disease load of HEV was calculated from nine of the 21 Global Burden of Disease (GBD) regions, amounting to around two-thirds of the world population. HEV-1 and HEV-2 resulted in around 20 million incident infections, with 3.4 million clinical cases causing 70,000 deaths and 3000 stillbirths [37]; however, several factors should be considered when evaluating these numbers Hepatitis E epidemics often occur on repeated occasions over several decades, within the same geographical region [3], with a disease frequency in these regions of approximately 6% [1]. Additionally, IgG anti-HEV antibodies show a dynamic response, with a sizeable proportion of the population showing a loss of antibodies over a period of 14 years [38]. Recently, HEV reinfections with altered immune response have been reported in a small number of patients [13,39,40]. Thus, these calculated numbers may grossly underestimate the disease load in such countries.

Diseases caused by HEV-1 and HEV-2 are self-limiting and chronic hepatitis and cirrhosis have not been reported [11,12]. Hepatitis E presents as a hyperendemic disease in low-resource countries with poor sanitation through fecal contamination of food and water supplies [2,3,41]. The epidemics occur at regular intervals within these communities, causing massive epidemics of jaundice and significant morbidity and mortality [4]. Regions with hyperendemic disease include most countries of southern Asia (India, Pakistan, Nepal, Bhutan, Sri Lanka, Nepal, and Bangladesh), southeast Asia (Indonesia, Cambodia, Thailand, Vietnam, Laos, and Burma), central Asia (Tajikistan, Kazakhstan, and Uzbekistan), north Africa (Sudan, Algeria, Tunisia, and Morocco), east Africa (Uganda, Kenya, and Burundi) and west Africa (Nigeria, Ivory Coast, Liberia, and Mali) [1]. Hyperendemic zones also include many countries in Latin America (Uruguay, Venezuela, Cuba, and Mexico) [42] and northwest China (Xinjiang Uyghur) [43]. Hepatitis E accounts for over half of acute hepatitis in these countries [15]. Disease is most often seen in young adults, and has high incidence and severity in pregnant women, oftentimes causing high maternal and perinatal mortality rates [8,10,16]. HEV superinfection in otherwise compensated cirrhotic patients commonly occurs and leads to hepatic decompensation and high mortality [44,45].

Hepatitis E presents as an endemic disease in developing countries with recent improvement in socioeconomic status and access to better sanitation and water supplies [46,47,48,49,50]. Hepatitis E in endemic zones causes around one-fourth of acute hepatitis and has all the clinical features of disease seen in hyperendemic zones except for epidemics of jaundice, yet it is only caused by the HEV-1 genotype. Hepatitis E is endemic in many countries of the Middle East (Turkey, Saudi Arabia, Yemen, Libya, Oman, Bahrain, Iran, Kuwait, and the United Arab Emeritus) and some regions of southeast Asia (Singapore).

HEV-1 is also the predominant cause of hepatitis in Egypt, which has a distinctive epidemiology of hepatitis E, resembling that of hepatitis A virus (HAV), with distinct subtypes not seen in the Asian population [51,52,53]. HEV exposure occurs in the community at a young age and most young adults have IgG antibodies to HEV. Notably, the increased incidence and severity of HEV infection in pregnant women is not seen in this community. In fact, HEV in pregnant women in Egypt is either asymptomatic or presents as mild disease.

### 3.2. HEV-3 and HEV-4

HEV-3 and HEV-4 infect humans and a number of other animal species, and are the main cause of zoonotic autochthonous sporadic infections [5,6,54]. Most autochthonous HEV diseases occur in older individuals. Such patients have a more severe liver disease, with higher hepatic or non-hepatic complications (15%) and acute liver failure (8%–11%), except for pregnant women, in which autochthonous HEV infections do not cause a severe disease. HEV superinfection in chronic alcoholics and alcoholic chronic liver disease has been reported, culminating in hepatic decompensation, progression of liver disease and an approximately 70% death rate. HEV-3 infections are known to cause chronic viremia, chronic hepatitis and cirrhosis in patients with solid organ transplants, HIV infections, and hematological neoplasms on chemotherapy, while HEV-4 is a self-limiting disease and does not cause chronic hepatitis and cirrhosis [55,56].

Autochthonous HEV-3 infection has global distribution. Apart from industrialized countries, it has been widely reported in several developing countries of Latin America (Argentina, Brazil, Bolivia, Cuba, Venezuela, Mexico, Uruguay, Chile, and Costa Rica), Russia, and northeast China. Autochthonous HEV-4 infections are the predominant infections encountered in China and several countries in Southeast Asia (Indonesia, Cambodia, Thailand, Vietnam, Laos, and Burma), with the exception of Japan.

## 4. Modes of Transmission

### 4.1. Waterborne Transmission

**Figure 1:**
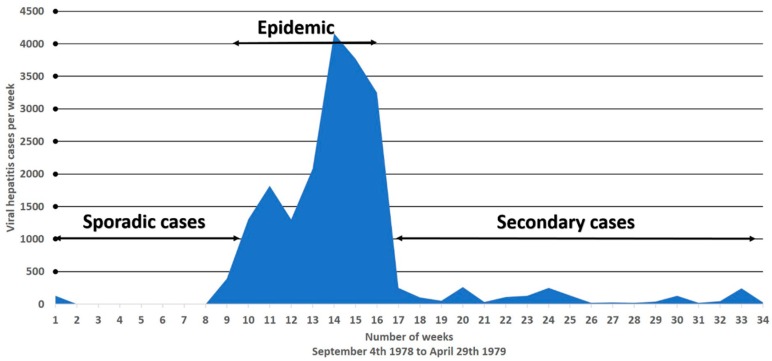
**Gulmarg Kashmir Epidemic, 1978–1979.** Weekly occurrence of 20,083 hepatitis E cases reported from 200 villages (*n* = 600,000) from 4 September 1978 to 29 April 1979 (34 weeks). Epidemic curve lasted from the 8th to the 17th week (nine weeks). Prior to and following the epidemic, only isolated cases of hepatitis E were recorded, suggesting that person-to-person transmission was not of major consequence to the development of the epidemic.

Hepatitis E is primarily transmitted through the fecal–oral route [3]. Gross fecal contamination of the community water supplies has been associated with several outbreaks in developing countries [8,57,58,59]. Epidemics are of common source origin as the epidemic curve is highly compressed, lasting for a duration of approximately six to eight weeks (Figure 1). Testing of water supplies at source and delivery points shows high *E. coli* counts, suggesting fecal contamination [8]. In several epidemics, there is a definite relationship between time of contamination and onset of disease, determined by the incubation period [57,58]. It has been shown that communities that use alternative water sources for drinking purposes (especially protected well water), prior to and during outbreaks, do not develop the disease [8]. Furthermore, raw sewage and water supplies have also been shown to contain HEV isolates closely related to human and animal infection [60,61,62,63].

**Figure 2:**
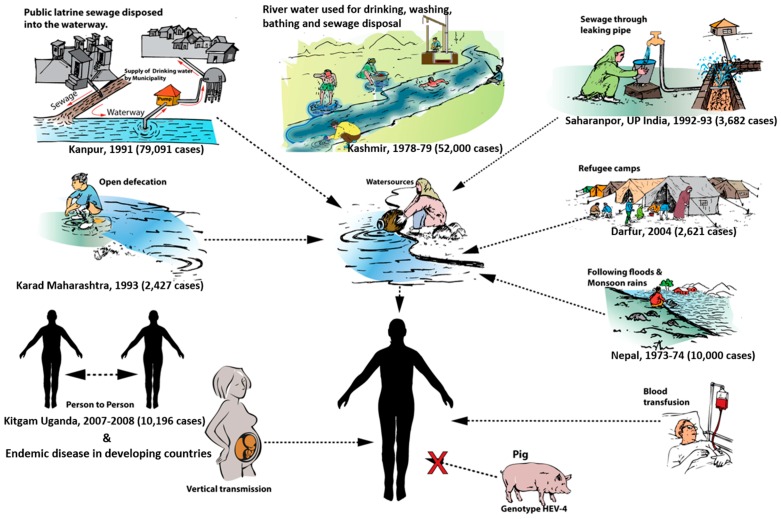
**Modes of transmission of hepatitis E in developing countries.** The settings for contamination of drinking water have been drawn in sketches, with epidemics reported in each case.

**Figure 3:**
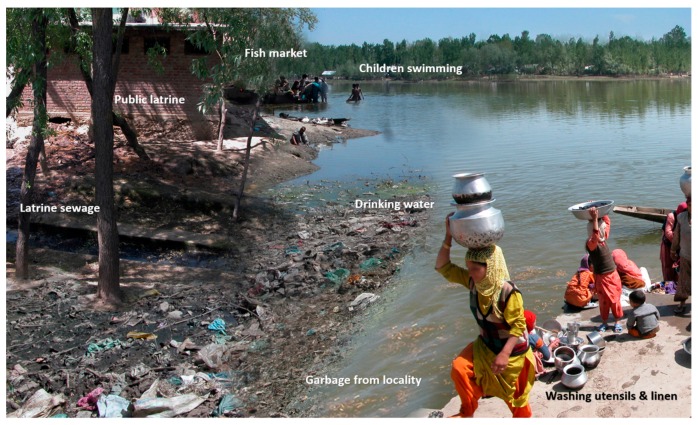
**Epidemic region: Kashmir, 1978.** Drinking water is collected from a canal in which public latrine sewage flows, garbage of the whole locality is dumped, utensils and linen are washed, children swim, and locals buy fish (Adapted from Khuroo, et al. [4]).

The mechanism of water contamination differs from one region to another; however, it typically follows a uniform pattern in repeated outbreaks within the same region, which is vital to understand, in order for public health officials to control any current and future epidemics [3]. Several different types of environmental settings have documented water contamination (Figure 2). Epidemics can result from contamination of river water used for drinking, washing, bathing, and sewage disposal (Figure 3) [4,39,64]. Outbreaks in such settings usually occur during winter months when the water level falls, thereby increasing the level of water contamination due to an increase in contaminant concentration [3]. Groundwater, crops, and waterways can all become contaminated. Open defecation in backyards and open fields can be another source of fecal contamination of groundwater, crops, and waterways [65]. In India for example, open defecation is rampant and over 300 million people use this practice for sanitary disposal, leading to widespread contamination of open drinking water sources, such as rivers, streams, and unprotected wells with raw sewage [2]. 

In fact, India accounts for 60% of individuals across the world, without access to toilets. It has been estimated that around 1.1 million liters of human feces are delivered to the Ganges River every minute. Flooding and monsoon rains wash fecally contaminated catchment areas like backyards, open fields, and ground water into open waterways, with a resultant eruption of waterborne diseases, including epidemics of hepatitis E [66].

Unfortunately, even piped water can become contaminated in certain situations. Quite often, piped drinking water is supplied to towns and cities in India, with the common practice of laying the pipes along with the sewage drains, or even crossing the sewage channels. Worn-out pipes become cracked and can start forming holes; during intermittent water supply schedules, the sewage enters the pipe lumen, causing fecal contamination of piped water. Several epidemics of hepatitis E in India have reportedly been caused by this phenomenon [67,68,69,70]. Even more dangerous is when the city sewage drains change their course due to heavy rains and flooding and enter the water source for the same city, thus supplying the city sewage water for drinking. Epidemics of massive proportion involving hundreds and thousands of cases have been reported due to sewage drain contamination of city water supplies alone [57,58].

**Figure 4:**
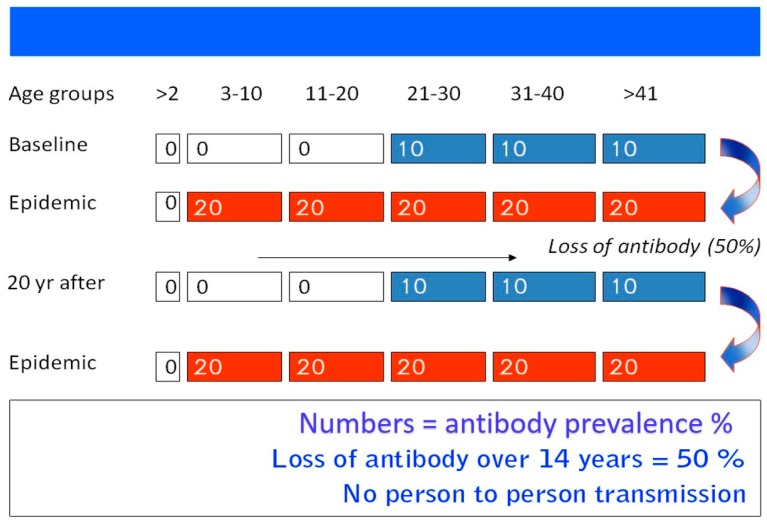
**Suggested model of repeated hepatitis E epidemics in hyperendemic areas.** IgG anti-Hepatitis E virus (anti-HEV) positivity in the general population is around 4%. Following the epidemic, around 20% are sero-positive. There is a general decline in sero-positivity of IgG anti-HEV and poor exposure to HEV infections during the inter-epidemic period. Repeat outbreaks occur when sero-positivity is around 4% and there is gross fecal contamination of drinking water sources (Adapted from Khuroo, et al. [4]).

One of the puzzling epidemiological features of hepatitis E is the fact that epidemics occur only periodically within the same geographical region, in spite of constant fecal contamination of water supplies (Table 1). For example, Kashmir, India has been under surveillance since the first epidemic of viral hepatitis was recorded and studied in 1978–1979. The first reported epidemic spread over 200 villages with 600,000 inhabitants; it amounted to 20,083 individuals with jaundice and 600 fatalities in a seven-week period (Figure 1). Initially, six epidemics occurred in adjacent regions on a yearly basis (1978 to 1984), all in the late autumn and early winter months when the fecal pollution of the waterways becomes concentrated, affecting a population of 1,937,000 individuals, and causing 53,307 cases of jaundice and 1752 deaths. Following this outbreak, a decade went by with no epidemics occurring within the population. However, in November of 2007, a second wave of epidemics was then recorded by Khuroo, et al. [39], who looked at the sero-epidemiology of this outbreak, comparing it to the first epidemic from 1978–1979. In 1993, 45 hepatitis E subjects infected in 1978–1979 were studied to determine the status of long-term IgG. Around half of the subjects with HEV infection had lost the antibodies over 14 years’ follow-up [38]. After a natural infection, IgG anti-HEV titers rise for four weeks, followed by a decline. Around 28%–67% of subjects have undetectable antibodies over a two-year follow-up [64,71]. Therefore, it is thought that periodic epidemics within a population is due to a cohort effect. During an epidemic, around one-fourth of the population becomes infected and show the presence of serum IgG anti-HEV. Following this, there is a gradual loss of antibodies over time and little exposure to HEV infection in the inter-epidemic period in a new cohort of the population. Once the herd immunity falls to a critical level, the next epidemic hits the community (Figure 4).

### 4.2. Person-to-Person Transmission

#### 4.2.1. Epidemic Disease

Transmission of HEV infection through person-to-person contact, causing epidemics, has long been a matter of controversy [72,73,74]. The epidemics are not followed by secondary waves of hepatitis cases [8,57] (Figure 1) and show poor intrafamilial spread [75]. These have been proposed as strong pieces of evidence that person-to-person transmission of HEV infections does not typically occur. Rather, as mentioned above, epidemics of hepatitis occur almost entirely due to gross fecal contamination of water, infecting all susceptible individuals, while secondary cases/intrafamilial spread are not expected to occur in this scenario due to a lack of susceptible individuals. Exceptionally, a large epidemic of hepatitis E that occurred in northern Uganda was related to person-to-person transmission [76,77]. This epidemic caused 10,196 icteric cases with 160 deaths; the epidemic curve was protracted, as new cases occurred from October 2007 through June 2008. There was a high attack rate in households, and 25% of cases occurring more than eight weeks after the onset of disease in the index case. Attending funerals, close contact with a jaundiced patient, and washing hands in a common family basin prior to having meals were all high risk factors for contracting the disease. These observations, along with an inability to define a common source of infection, suggest that the epidemic spread through person-to-person contact. Several outbreaks recorded a more protracted epidemic curve, and/or multiple epidemic peaks, which suggest alternative routes for the spread of infection. However, a detailed epidemiological assessment as to the mode of the spread of disease was not conducted [4,58]. Therefore, it is possible that family sharing of utensils for hand washing and drinking and eating, a common practice in resource-poor countries, along with poor hand hygiene, may allow cross-contamination of water and eatables, leading to person-to-person transfer of the virus during an outbreak.

#### 4.2.2. Sporadic Disease

The mode of transmission of sporadic disease caused by HEV-1 and HEV-2 is also under scrutiny [72]. In the first reported series on sporadic hepatitis E, it was found [15] that 51 (33%) of the 155 cases had had recent contact with another case of jaundice, suggesting that person-to-person contact could play an important role in the transmission of sporadic hepatitis E. Khuroo, et al. [74] studied 62 household contacts of 13 index patients and showed that 18 (29%) had developed evidence of HEV infection at 31 ± 4.5 days after the onset of disease in the index patient. This suggested that the disease was spread via household contact from the index patient, rather than simultaneous infection of the index patient and household contact. However, Somani, et al. [78] reported that intrafamilial spread of sporadic HEV infection occurred infrequently. It is possible that sporadic HEV infections may occur in the setting of environmental contamination of agricultural products and water resources, and spread of infections through infected food and water. Raw sewage has been found to contain infectious HEV strains in developing countries, which closely relates to human HEV-1 and HEV-2 [61,79,80].

### 4.3. Zoonotic Transmission

**Figure 5:**
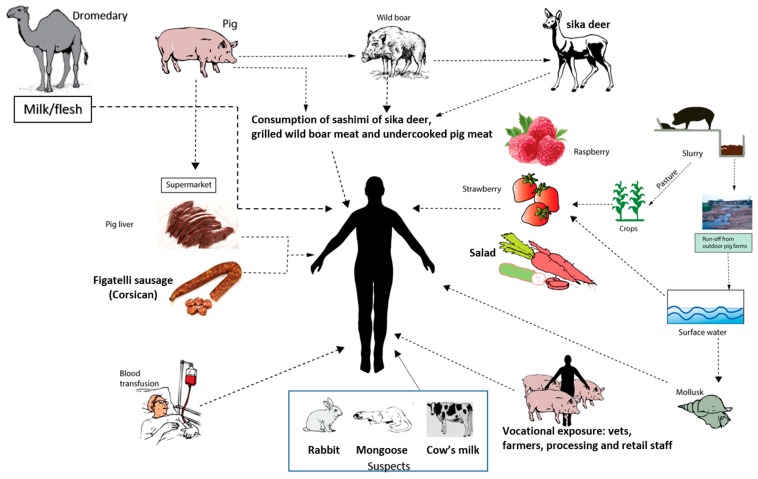
Zoonotic transmission of hepatitis E in developed and many developing countries.

Hepatitis E is a zoonotic disease, and domestic pigs, wild boar, and sika deer are reservoirs for genotypes HEV-3 and HEV-4 [81]. Human infections occur through three methods, namely, zoonotic foodborne consumption, direct contact with infected animals, and environmental contamination by animal manure run-off (Figure 5) [82]. Food-borne zoonotic transmission of HEV-3 and HEV-4 has been well studied [56,82]. Wild boar, sika deer, and domestic pigs cross transmit HEV [83], and eating the parboiled flesh or liver (a delicacy in many countries) could be responsible for the autochthonous cases and outbreaks of hepatitis E [84]. A more common method of the spread of HEV is through consumption of raw livers from supermarkets or eating Corsican figatelli sausage in Europe [85,86,87]. Such livers and sausages are often infected with live HEV. Vocational exposure to domestic pig farms, manure, and sewage is also a significant risk factor in HEV infections in many countries [88,89,90]. Swine veterinarians as well as workers, were found to be 2 to 5 times more likely to be positive for IgG anti-HEV than non-swine veterinarians and the general population in many European countries [91,92,93,94]. Additionally, pig slurry can cause environmental contamination through several ways. Use of pig slurry as pasture can infect agricultural products like raspberries, strawberries, and many vegetables used in salad [95,96]. Run-off from outdoor pig farms causes contamination of surface water as well as produce receiving surface water [97,98]. 

Run-off from outdoor pig farms can also contaminate coastal waters and seafood, such as fish and shellfish [99]. HEV has been identified in commercially available mussels from several European countries [100,101], Korea [102], and Japan [103]. Consumption of shellfish has been identified as a risk factor for hepatitis E in several case reports [104,105,106] and in an outbreak of hepatitis E aboard a cruise ship [107].

HEV infections in animals in India have been the focus of a number of studies. HEV infection is ubiquitous in several animal species, including domestic pigs, sheep, goats, and buffalo [108]. Arankalle, et al. [109] studied 284 domestic pigs from Maharashtra, India were studied, where 122 (42.6%) and 13 (4.6%) animals were reactive for IgG antibodies to HEV and HEV RNA, respectively. All isolates from domestic pigs were of genotype HEV-4. Therefore, since human HEV infections are caused uniformly by genotype HEV-1 in India, both epidemic and sporadic, it is believed that human and pig HEV infections are unrelated, and zoonotic transmission plays an insignificant role in human infections in India [109].

While studying HEV in dromedaries in Dubai, U.A.E., Woo et al. [31] used fecal samples to isolate from three camels a previously unrecognized HEV genotype, designated as HEV-7. Subsequently, a liver transplant patient from the Middle East was found to be infected with HEV-7 and had developed chronic hepatitis, requiring antiviral therapy. He regularly consumed camel flesh and drank camel milk [30]. Recently 2438 dromedary samples from Sudan, Somalia, Egypt, Kenya, Pakistan, and the United Arab Emirates were screened for HEV-7. Ninety-six (45.7%) of the 210 animals were seropositive for IgG anti-HEV, while HEV RNA was isolated from 12 (0.5%) of the 2171 serum samples and five (1.9%) of the 267 fecal samples [110]. Further studies need to be done to evaluate the potential of camelid HEV-7 to cause the human disease.

Hepatitis E in China has a complex and changing epidemiology. Initially, China was a hyperendemic zone for HEV-1, and contained multiple epidemics of hepatitis E [111]. In fact, China witnessed the largest ever (119,280 cases) epidemic of hepatitis E from 1986 to 1988 [112]. However, HEV-4 has now become the dominant genotype infecting the Chinese population. This is especially true for northeast China and is possibly related to the introduction of infection via animal reservoirs, especially domestic pigs [113].

### 4.4. Parenteral Transmission

In a case control study, Khuroo, et al. [114] documented transfusion-transmitted hepatitis E. Thirteen HEV infections (IgM anti-HEV & HEV RNA) of 145 multiply transfused subjects were detected, as opposed to two infections of 250 subjects not transfused. 25 susceptible (IgG anti-HEV negative) patients were prospectively studied following multiple transfusion, and three post-transfusion infections were detected. These infections were traced to four of the 107 donors. These donors were positive for HEV RNA at the time of blood donation. All four donors were asymptomatic and all transfusion-associated infections in Kashmir were caused by genotype HEV-1. Subsequent to this report, a Japanese patient was found to have developed transfusion-associated HEV infection and the authors reported a complete donor–patient sequence homology, confirming observations on transfusion-transmitted hepatitis E [115]. Several cases and case series of transfusion-associated HEV infections were reported from multiple countries [116,117]. In a recent study by Hewitt, et al. [118], 18 (42%) of 42 HEV-positive transfusion recipients were found to have contracted HEV-3 infections. Of these, three had short-lasting viremia, while 10 developed prolonged and persistent infections.

HEV RNA has been detected in healthy populations from many countries (Table 2) [119,120,121]. Viremia varied from 1 out of 672 donations (Germany) to 1 out of 8416 donations (Austria). The duration of viremia extends up to 45 days and the infectious dose of HEV is as low as its detection by RT-PCR [122,123,124,125]. Based on these data, it was estimated that around 80,000–100,000 transfusion-associated HEV infections had occurred in England in 2013. Of the 7.4 million blood products administered in Germany per year, 1600 to 5900 transfusion-associated HEV infections had occurred. Blood or blood products are often required for several clinical conditions in which hepatitis E runs a more severe course or leads to chronic hepatitis and cirrhosis. These include pregnancy, liver disease, solid organ transplant (SOT), HIV positive status, and hematological neoplasm. In view of the above data, there is a need to conduct screening of blood donors in countries with high HEV prevalence.

### 4.5. Vertical Transmission

HEV can be transmitted vertically to the fetus and infant from infected mothers. We studied eight mothers who had HEV infection for evidence of vertical transmission. Six babies had contracted vertically transmitted HEV infection [14]. In another study, 26 pregnant women with HEV infection were studied for a pattern of vertically-transmitted HEV infection. [130]. Five mothers had died prior to delivery, two aborted the fetuses, four delivered premature babies, and 15 had completed pregnancy with normal deliveries. Out of the 19 babies evaluated, 12 were reactive for IgM anti-HEV and 10 showed viremia. In all, 15 (78.9%) had evidence of intrauterine HEV infection. HEV infection in babies presented as icteric hepatitis in seven, anicteric hepatitis in five, and jaundice alone in three. Six newborns died from liver failure and one neonate died from premature birth and low birthweight. Nine HEV infected babies who survived had short-lasting viremia and none showed evidence of chronic hepatitis or cirrhosis. Subsequent to these reports, a number of reports have documented intrauterine transmission of HEV infection with high maternal and fetal mortality (Table 3) [50,131]. Recently, HEV replication was found to occur within the placenta and was correlated with fetal and maternal mortality from acute liver failure [132]. In a study by Khuroo, et al. [133], the outcome of 36 pregnant women with hepatitis E was evaluated, assessing the occurrence and severity of vertically transmitted HEV infection in fetuses/neonates, and a relationship between the severity of disease in the fetus with the severity and outcome of disease in the mother was found. This lead to the postulation that acute liver failure in pregnant women may be an example of mirror syndrome, akin to acute fatty liver of pregnancy.

### 4.6. Nosocomial Transmission

Nosocomial transmission of HEV infections is unusual and most healthcare workers and inpatient populations are not at a high risk for contracting the virus. However, several reports have focused on the transmission of HEV infection in hemodialysis units [137,138,139,140] and a few have reported outbreaks of hepatitis E in hospital patients and healthcare workers [141,142,143].

### 4.7. Sexual Transmission

There is no direct evidence that HEV is transmitted by sexual contact. However, several studies have shown a higher seroprevalence of IgG anti-HEV in homosexual men and those infected with HIV [144,145,146,147,148].

### 4.8. HEV and Milk

Chibber, et al. [135] studied 93 HEV infected mothers for HEV RNA in colostrum and the role of breast feeding to transmit infection to neonates. Anti-HEV antibody and HEV RNA were uniformly detected in colostral samples of HEV-infected mothers. However, the levels of antibodies and HEV RNA were significantly lower than those in maternal blood samples, and therefore breastfeeding was considered to be an unlikely route of transmission for neonatal HEV infection [135]. Recently however, HEV RNA was isolated from breast milk from a mother with acute HEV [149]. Subsequently, additional studies need to be performed to test breast milk for HEV RNA and advise whether neonates should be breastfeeding from infected mothers.

As mentioned above, transmission of camelid HEV (genotype 7) from a dromedary to a liver transplant patient was reported in Dubai [3,4]. This patient had regularly consumed camel flesh and camel milk [30]. As HEV infections are common in dromedaries from many countries, the role of camel milk in the spread of HEV in such communities needs further scrutiny [110]. Recently, Huang, et al. [150] studied 140 cows from Dali, Yunnan, China, from September to December 2015. In this community, mixed farming of domestic animals is a common practice. Active HEV infection was found in 52 cows, as determined by viral RNA positivity in fecal samples. All 52 infected animals were excreting HEV-RNA in milk samples in high titers. HEV-RNA recovered from raw and pasteurized milk samples have also been shown to transmit the disease to rhesus monkeys. However, a short period of boiling, even without pasteurization, has the potential to completely inactivate HEV. Phylogenetic analysis revealed that all HEV isolates from cow-milk belong to genotype 4 and subtype 4h. These findings need to be confirmed, and the role of infectious HEV-contaminated cow’s milk in the spread of HEV infections in endemic regions must be further defined.

## 5. Conclusions

In conclusion, epidemics of hepatitis E caused by HEV-1 and 2 in resource-poor countries are spread through water that has been contaminated by sewage, of which, several environmental settings contribute to fecal contamination of water supplies to such communities. Person-to-person spread of HEV infection has been implicated in epidemics with a protracted course and also in the endemic disease. Food-borne zoonotic transmission is the dominant route of spread for HEV-3 and -4 and occurs through the consumption of parboiled meat or liver of HEV-infected domestic pig, wild boar, sika Deer or Corsican figatelli sausage. The waste water of domestic pig dung in such countries may pollute waterways and infect those who visit sea beaches or ingest infected mollusks. HEV infection in pregnant women is uniformly spread through vertical transmission to the fetus, which can cause significant morbidity and mortality in fetuses and newborn babies. Transfusion-transmitted HEV is now being recognized as an important route of transmission in many countries and poses a potential risk to organ transplant patients. Lastly, recent data on the spread of HEV-7 through camel flesh/milk and detection of HEV-4 in high titers in cow’s milk in mixed animal farms from China, opens up a new vista for exploring the transmission routes of HEV infection.

## Figures and Tables

**Table 1:** Account of 10 periodic epidemics recorded in Kashmir, India from 1978 to 2013.

Place	Year	Population Exposed	Icteric Cases	Deaths
Gulmarg	1978–1979	600,000	20,083	600
Sopore	1979–1980	200,000	6000	200
Handwara	1980–1981	400,000	11,500	400
Jammu Army Camp	1981–1982	845	206	0
Kupwara	1981–1982	500,000	15,000	550
Jammu	1983–1984	176,833	518	2
Pinglina	1993–1994	10,000	156	2
Shopian	1994–1995	60,000	1500	17
Maharajpora, Sopore	2007–2008	720	21	2
Pattan, Gulmarg	2012–2013	20,000	600	2
**Total**	1978–2013	1,968,398	55,563	1775

**Table 2:** HEV RNA in blood donors.

Reference	Region	HEV RNA (%)	HEV Infected Transfusion vs. Total Donations
Arankalle et al., 2000 [120]	Pune India	3/200 (1.5%)	1:67
Khuroo et al., 2004 [114]	Kashmir India	4/107 (3.7%)	1:27
Gotanda et al., 2007 [126]	Japan	9/6700 (0.13%)	1:745
Ren et al., 2013 [127]	China	6/10,741 (0.06%)	1:1790
Juhl et al., 2014 [125]	Germany	35/23,500 (0.14%)	1:671
Hewitt et al., 2014 [118]	England	79/225,000 (0.04%)	1:2848
Hogema et al., 2014 [128]	Netherlands	20/35,220 (0.06%)	1:1761
Fischer et al., 2015 [129]	Austria	7/58,915 (0.01%)	1:8416

Infectious dose required for HEV infection seems to be low. Duration of viremia in asymptomatic donors does not exceed 45 days (Adopted from Khuroo, et al. [1]).

**Table 3:** Vertical transmission of hepatitis E from mother to fetus.

Reference	Region	Mothers	Vertical Transmission (%)	Deaths
Khuroo, et al., 1995 [14]	Kashmir, India	8	6 (75%)	3 (50%)
Kumar, et al., 2001 [50]	Al-Khobar, KSA	26	26 (100%)	2 (7.7%)
Kumar, et al., 2004 [134]	New Delhi, India	18	6 (33.3%)	-
Singh, et al., 2003 [131]	New Delhi, India	6	3 (50%)	-
Chibber, et al., 2004 * [135]	Al-Khobar, KSA	6	4 (66.6%)	-
Khuroo, et al., 2009 [130]	Kashmir, India	19	15 (78.9%)	-
Zaki, et al., 2013 [136]	Mansoura, Egypt	9	6 (66.6%)	-

* All 57 HEV-infected mothers had low levels of HEV RNA in the colostrum.
